# Electrically Conductive, Transparent Polymeric Nanocomposites Modified by 2D Ti_3_C_2_T_x_ (MXene)

**DOI:** 10.3390/polym11081272

**Published:** 2019-07-31

**Authors:** Aisha Tanvir, Patrik Sobolčiak, Anton Popelka, Miroslav Mrlik, Zdenko Spitalsky, Matej Micusik, Jan Prokes, Igor Krupa

**Affiliations:** 1 Center for Advanced Materials, Qatar University, P. O. Box 2713, Doha, Qatar; 2 Centre of Polymer Systems, University Institute, Tomas Bata University in Zlin, Trida T. Bati 5678, 760 01 Zlin, Czech Republic; 3 Polymer Institute Slovak Academy of Sciences, Dubravska Cesta 9, Bratislava 845 41, Slovakia; 4 Charles University Prague, Faculty of Mathematics and Physics, V Holešovičkách 2, 182 00 Prague 8, Czech Republic

**Keywords:** polymer-matrix composites (PMCs), MXene, electrical properties, casting

## Abstract

The electrically conductive, transparent, and flexible self-standing thin nanocomposite films based on copolyamide matrix (coPA:Vestamelt X1010) modified with 2D Ti_3_C_2_T_x_ (MXene) nanosheets were prepared by casting and their electrical, mechanical and optical properties and then, were investigated. The percolation threshold of the MXene filler within the coPA matrix was found to be 0.05 vol. %, and the highest determined electrical conductivity was 1.4 × 10^−2^ S·cm^−1^ for the composite filled with 5 wt. % (1.8 vol. %) of MXene. The electrical conductivity of the as-prepared MXene was 9.1 S·cm^−1^, and the electrical conductivity of the MAX phase (the precursor for MXene preparation) was 172 S·cm^−1^. The transparency of the prepared composite films exceeded 75%, even for samples containing 5 wt. % of MXene, as confirmed by UV spectroscopy. The dynamic mechanical analysis confirmed the improved mechanical properties, such as the storage modulus, which improved with the increasing MXene content. Moreover, all the composite films were very flexible and did not break under repeated twisting. The combination of the relatively high electrical conductivity of the composites filled with low filler content, an appropriate transparency, and good mechanical properties make these materials promising for applications in flexible electronics.

## 1. Introduction

Materials that are flexible, optically transparent and electrically conductive are of paramount interest in numerous applications, such as wearable electronics and touch-sensitive screens [1,2,3]. Traditionally, indium tin oxide has been used as the standard material for such applications; however, its high cost and nonflexible nature has encouraged the search for alternative materials [4,5]. As a result, various conductive nanofiller/polymer composite materials have been prepared [6,7,8]. Many conductive materials have been studied, including graphene [9,10], carbon nanotubes [11,12], carbon fibers [13], metal particles [14,15], ceramic particles [16] and conductive polymers [17,18].

MXenes are a relatively new family of (2D) transition metal carbides, nitrides or carbonitrides [19,20], first reported in 2011 by the Gogotsi and Barsoum groups [21]. MXenes are produced by the selective chemical etching of A from a MAX phase, where M is a transition metal, A is a group IIIA or IVA element and X is C or N. These materials have received tremendous attention from the scientific community due to their excellent physiochemical properties, electrical conductivity and hydrophilicity [22]. These properties make them promising candidates for use as electrodes for electrochemical supercapacitors [23,24,25], catalytic promoters [26,27], absorbents for heavy metal ions [28], materials for the photodegradation of dyes [29], (bio)sensing [30,31], and anti-biofouling membranes [32,33]. Among several MXenes reported in the literature, Ti_3_C_2_T_x_, which is obtained by etching Al layers from Ti_3_AlC_2_, has been explored the most intensively [25,28,34,35].

Due to the advantageous physical properties of MXenes, specifically their mechanical strength, electrical conductivity and thermal conductivity, various polymer composites based on MXenes have been investigated. For instance, An et al. [36] realized MXene coatings of stretchable Polydimethylsiloxane that can withstand a large-scale mechanical deformation while maintaining an electrical conductivity as high as 20 S·cm^−1^. Feng et al. [37] prepared the sandwich Polyvinylidene fluoride (PVDF) composite and demonstrated improved electrical properties. The permittivity of ~26 at 100 Hz, a breakdown strength of ~350 MV·m^−1^ and AC conductivity 10^−11^ S·cm^−1^ at 100 Hz was reported. Sun et al. [38] coated polystyrene microspheres with MXene nanosheets. They observed that the particles’ diameter plays a critical role in determining the electrical conductivity of the Ti_3_C_2_T_x_/PS nanocomposites with the highest value of conductivity of 11 S·cm^−1^. Very high electric conductivities can be also obtained in composites based on conductive polymers. The electrical conductivity of poly(3,4-ethylenedioxythiophene) polystyrene sulfonate composites was 1489 S·cm^−1^ (9:1 MXene: polymer) [39] or 340 S·cm^−1^ (7:1) [40]. The electrical conductivity of Ti_3_C_2_T_x_/PVA films (90/10 w/) was found to be of 224 S·cm^−1^ [41]. The mechanical properties of ultrahigh molecular weight polyethylene and MXene composites showed improved mechanical properties, particularly the surface hardness and creep performance [42]. Polyvinyl alcohol has also been employed [43], and PVA-MXene composite nanofibers with concentrations as low as 0.14 wt. % Ti_3_C_2_T_x_ exhibited a DC conductivity of 0.8 × 10^−3^ S·cm^−1^. PVDF [44] was used to create PVDF-MXene composites, which exhibited an approximately 1-fold enhancement in thermal conductivity compared with neat PVDF, accompanied by a pronounced increase in the dynamic mechanical properties of the PVDF, such as enhanced storage moduli and shifted *T_g_* towards higher temperatures with increased MXene loading. The poly(vinylidenefluoride-trifluoro-ethylene-chlorofluoroehylene) polymer filled with 2D Ti_3_C_2_T_x_ nanosheets reached a dielectric permittivity as high as 10^5^ near the percolation limit of approximately 15.0 wt. % MXene loading [45]. Polydiallyldimethylammonium chloride (PDDA) [41] was used to synthesize PDDA composites, which also showed capacitance values of 528 F·cm^−3^ at 2 mV·s^−1^ and 306 F·cm^−3^ at 100 mV·s^−1^ with good cyclability. Polyacrylamide (PAM) [46] was used for a PAM/MXene composite, and these materials showed an electrical conductivity of 3.3 × 10^−4^ S·cm^−1^ with only a 6 wt. % (1.7 vol. %) MXene loading. 

The presented study is specifically focused on the preparation and characterizations of electrically conductive, transparent and flexible polymeric composites based on MXene-filled hotmelt type of coPA-6,10 that were prepared by a simple casting method to form self-standing films. The prepared composites were characterized by energy-dispersive X-ray spectroscopy (EDS) and X-ray diffraction (XRD) to confirm good dispersion of the MXene particles within a coPA matrix. The dielectric (BDS), dynamic mechanical analysis (DMA) and ultraviolet spectroscopy (UV spectroscopy) studies were performed to evaluate the performance of the prepared composites. The composites exhibited good electrical conductivity and a high transparency and mechanical performance, which would support the use of these materials in many applications, such as sensors and screens.

## 2. Materials and Methods 

### 2.1. Materials

CoPA (Vestamelt X1010, EVONIK Industries, Marl, Germany), propanol (Sigma Aldrich, St. Louis, MO, USA), Ti_3_AlC_2_ (Y-Carbon, Ltd., Kiev, Ukraine), and hydrofluoric acid (Sigma Aldrich, St. Louis, MO, USA) were used to prepare the samples. All other solvents and reagents were analytical grade obtained from Sigma Aldrich (St. Louis, MO, USA) and were used without further purification.

### 2.2. Preparation coPA/MXene Composites

The Ti_3_C_2_T_x_ (MXene) sheets were prepared by a conventional hydrofluoric acid (HF) etching protocol, as described earlier [44]. The prepared MXene was diluted in water and subsequently delaminated by sonication in a bath sonicator for 60 min. Then, the delaminated MXene dispersion was centrifuged at 3500 rpm for 45 min, and the collected supernatant was dried, which led to delaminated MXene particles.

To prepare the polymer composites, the dried MXene was dispersed in propanol using a probe sonicator (Hielscher 400S with Sonotrode H7, Teltow, Germany) for 20 min with an amplitude of 80% at 0.50 s per cycle, and the coPA was subsequently added to obtain a 10 wt. % solution. The concentration of MXene was varied from 0.5 to 5 wt. % with respect to the concentration of coPA. The composite films were prepared by film casting using a Pro-cast machine (HED International Inc., Ringoes, New Jersey, USA). Typically, 10 mL of solution was used for one batch. The tape thickness was 0.5 mm, the tape width was 90 mm, and the conveyor speed was 30 mm per minute. The temperature of the furnace was set at 80 °C. The prepared films were peeled from the plastic support and dried for 24 h at room temperature and subsequently in a vacuum oven at 60 °C for 4 h to remove any remaining solvent.

### 2.3. Characterization

The surface morphology of the specimens was examined with field emission scanning electron microscopy (FE-SEM, Nova Nano SEM 650, Memphis, TN, USA) equipped with energy-dispersive X-ray spectroscopy (EDS) (Memphis, TN, USA) with secondary electron images at 3 kV and different magnifications. All specimens were sputter-coated with 2 nm of gold before SEM images were taken.

The morphology of the neat powder and cross-section of the composite with 5 wt. % of MXene particles was investigated using transmission electron microscopy (TEM, JEM-2100Plus, JEOL, Peabody, MA, USA). Powder MXene was dispersed in distilled water to obtain a 0.05 wt. % dispersion. Then, a drop of dispersion was deposited directly on the TEM grid and evaporated under ambient conditions. A cross-section of the composite was obtained using a microtone device with liquid nitrogen.

Atomic force microscopy (AFM) was used to measure the thickness of MXene particles using MFP3D Asylum research (Santa Barbara, CA, USA) equipped with a Silicon probe (Al reflex coated Veeco model–OLTESPA, Olympus; spring constant: 2 N m^‒1^, resonant frequency: 70 kHz). The measurements were performed under ambient conditions using the standard topography AC air (tapping mode in air). An AFM head scanner applied with Si cantilever adjacent vertically in the sample resonant frequency of the free-oscillating cantilever was set as the driving frequency. The specimen was prepared by deposition of one drop of 0.05 wt % of MXene in ethanol onto silicon substrate wafer and dried at 60 °C overnight as the prepared specimen was used for AFM measurement. 

The X-ray diffraction (XRD) analysis was performed on a Bruker D8 ADVANCE X-ray diffractometer (Bruker Corp., Billerica, MA, USA) equipped with Cu Kα radiation (λ = 0.154 nm). The scanning range (2θ) was from 5° to 60° at a scan speed of 2°/minute. UV spectra were recorded with a spectrometer (SEC2000-UV/VIS, ALS, Tokyo, Japan) using a quartz cuvette. The data were obtained at a constant bandpass with a resolution of 2 nm.

The average particle size and size distribution were determined by laser light scattering with a Malvern Zetasizer (NANO-ZS, Malvern Instruments Ltd., Worcestershire, UK) in a polystyrene cell at room temperature. The measurement was repeated twice. A volume of 10 mL of the MXene dispersion in distilled water (1 mg/mL) was prepared by sonicating with an ultrasonic tip (Hielscher 400S with Sonotrode H7) for 20 min with an amplitude of 80% at 0.50 s per cycle.

The X-ray photoelectron spectroscopy (XPS) data were recorded using a Thermo Scientific K-Alpha XPS system (Thermo Fisher Scientific Inc., East Grinstead, UK) equipped with a microfocused, monochromatic Al K X-ray source (1486.68 eV). An X-ray beam of 400 µm size was used at 6 mA and 12 kV. The spectra were acquired in the constant analyzer energy mode with a pass energy of 50 eV for the narrow regions. Charge compensation was achieved with the system flood gun. Thermo Scientific Advantage software, version 5.9904 (Thermo Fisher Scientific Inc., Waltham, MA, USA), was used for digital acquisition and data processing. Spectral calibration was achieved using the automated calibration routine and the internal Au, Ag, and Cu standards supplied with the K-Alpha system. The surface compositions (in at %) were determined by considering the integrated peak areas of the detected atoms and the respective sensitivity factors. The fractional concentration of a particular element A was computed using Equation (1):

(1) $$\%A=\frac{Is/sn}{\Sigma \left(\frac{Is}{sn}\right)}\times 100\%$$

where I*n* and s*n* are the integrated peak areas and the Scofield sensitivity factors corrected for the analyzer transmission, respectively.

The dynamic mechanical analysis was conducted using an RSA-G2 (TA Instruments, New Castle, DE, USA) in tensile mode at 25 °C under an air atmosphere. The rectangular samples (40 mm × 6 mm × 0.02 mm) were prepared and measured in the linear viscoelastic range. The mechanical response was investigated in the temperature range of 30–150 °C with a strain deformation of 0.01% and at a frequency of 1 Hz with a 5 °C/min heating rate. 

The DSC measurements were performed using a Perkin Elmer model DSC 8500 (Perkin Elmer, Waltham, MA, USA) over a temperature range from 20 to 220 °C at a heating rates of 3 and 20 °C/min under nitrogen atmosphere. Nitrogen gas was passed through the instrument at a flow rate of 20 mL/min. The weight of the samples varied from 7 to 8 mg.

The dielectric measurements were performed using a Novocontrol GmbH Concept 40 broadband dielectric spectrometer (Montabaur, Germany), and the data were collected over the frequency range of 0.1 Hz ± 3 MHz at fixed temperatures in the range of 150 to 100 °C. The sample discs with diameters of 2 cm were sandwiched between two gold-coated copper electrodes with a 2 cm diameter and then transferred to the instrument for data collection.

The samples used for electrical conductivity measurements were prepared from powders by compressing them at 530 MPa into 13 mm diameter pellets with a thickness of approximately 1 mm.

The electrical conductivities of the original MAX phase and the synthesized MXene were determined by a four-point method in a van der Pauw arrangement using a Keithley 220 Programmable Current Source, a Keithley 2010 Multimeter as a voltmeter and a Keithley 705 Scanner equipped with a Keithley 7052 Matrix Card. Two principal limits must be taken into consideration to choose the correct measuring current. The low current causes low precision of the voltage reading, while high values of the current can damage the contact area between the metal contact and the sample surface due to Joule heating (a small contact area results in a high current density). The specific densities of the coPA and MXene were determined at room temperature using a pycnometer, and the values were found to be 1.11 g·cm^−3^ for coPA and 3.57 g·cm^−3^ for MXene.

## 3. Results and Discussion 

### 3.1. MXene Characterization

**Figure 1:**
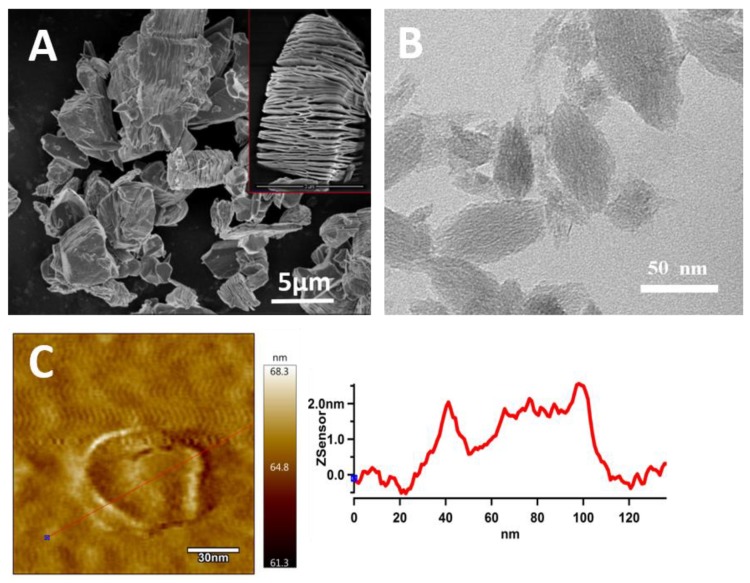
(**A**) SEM of MAX phase and (**B**) TEM image and (**C**) atomic force microscopy (AFM) image with line profile of MXene particles.

A treatment of the MAX phase by HF caused exfoliation of the MXene layers, as clearly demonstrated in Figure 1A. The successful delamination of the prepared MXene was performed by sonication, and the TEM of MXene particles is shown in Figure 1B. The particles of MXene were delaminated into the individual layers and had 2D shapes with lengths from 50 to 100 nm and widths from 20 to 40 nm.

The thickness of the nanosheets has been determined by AFM as seen in Figure 1C,D. AFM shows individual MXene nanosheets. The thickness of 1.8 ± 0.3 nm was determined from five individual measurements which is in line with observations from other studies [47].

**Figure 2:**
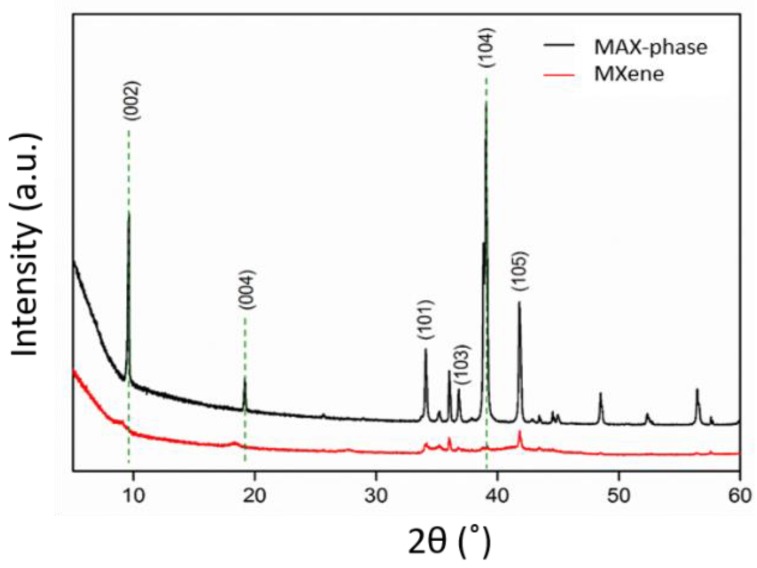
X-ray diffraction (XRD) of MAX phase and MXene particles.

XRD was performed to confirm the successful preparation of MXene from the MAX phase through the removal of Al between the layers (Figure 2). As reported in the literature [3], the characteristic (002) peak of Ti_3_AlC_2_ at 9.5° broadens and shifts to a lower value, indicating the removal of Al and subsequent structural expansion due to the substitution of Al with –F and –OH/O terminating groups, resulting in a larger d-spacing. Similarly, most of the non-basal plane peaks of Ti_3_AlC_2_, most notably the most intense peak at 39°, disappeared. On the other hand, the (001) peaks, such as the (002), (004), (101), and (103) peaks, broadened, decreased in intensity and shifted to lower angles compared to their locations before etching, which was mainly caused by removing Al from the MAX phase [48].

Table 1 summarizes the surface chemical composition of the initial MAX phase and prepared MXene calculated from the deconvolution of particular high-resolution spectra. The surface of the MAX phase and MXene was partially oxidized, which demonstrates the presence of a Ti2p signal at ~459 eV corresponding to TiO_2_ and an Al2p signal at ~74.8 eV corresponding to AlOx, most likely Al_2_O_3_ [49]. After etching of the MAX phase by HF, there is a clear decrease in the aluminum content, but some Al still remained in the MXene structure, such as Al carbide (~70.5 eV) and Al_2_O_3_ (~74.8 eV). In MXene, a higher Ti carbide content (C1s at ~281.6 eV, 9.6 at. % and Ti2p at ~454.7 eV, 6.8 at. %) indicates the development of the Ti_2_C_3_ structure.

**Figure 3:**
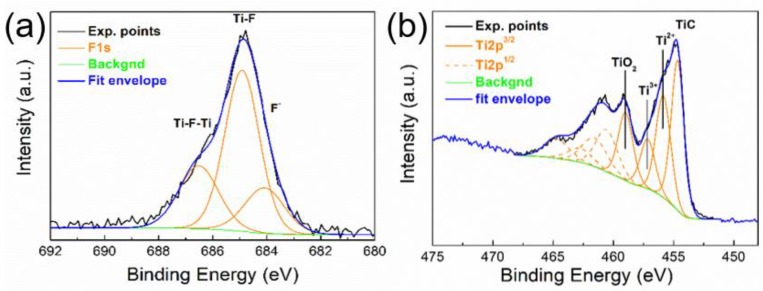
XPS (**a**) Ti2p and (**b**) F1s region of MXene.

In addition to the signal of TiO_2_ and Ti_2_C_3_, Ti2p in the case of MXene also exhibits an increase in the signal at ~455.9 eV (labeled as Ti^2+^) and at ~457.2 eV (labeled as Ti^3+^) (Figure 3a). These signals, in addition to some oxidation, might also be correlated with fluorine binding, which is readily embedded in the structure of the prepared MXene. The MXene F1s spectrum (Figure 3b) has a strong signal at ~684.9 eV, which can correspond to fluorine binding to the Ti carbide and creating an F–Ti–C bond. The F1s signal at a slightly higher binding energy of ~686.5 eV might even indicate a fluorine bridging atom (C–Ti–F–Ti–C) [50].

### 3.2. Composite Characterization

#### 3.2.1. MXene Dispersion

**Figure 4:**
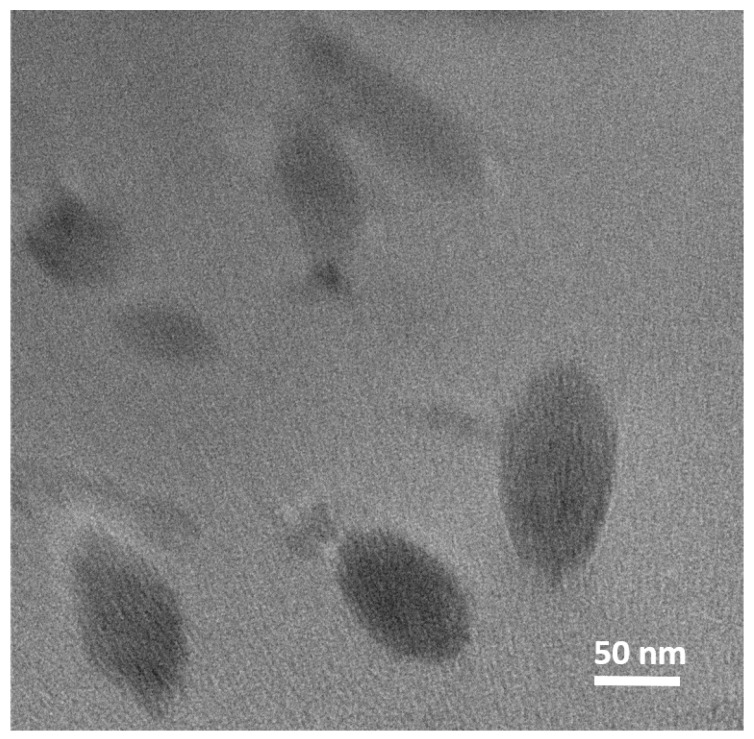
TEM image of the cross-section of the composite with 5 wt. % of MXene particles.

As shown in Figure 4, MXene particles mixed with the polymer matrix were sustained in the exfoliated state and did not show any significant agglomeration. The particles were well-dispersed, as supported by the size of the MXene particles within the matrix, which remained similar to the size obtained from TEM measurements (Figure 1b)

**Figure 5:**
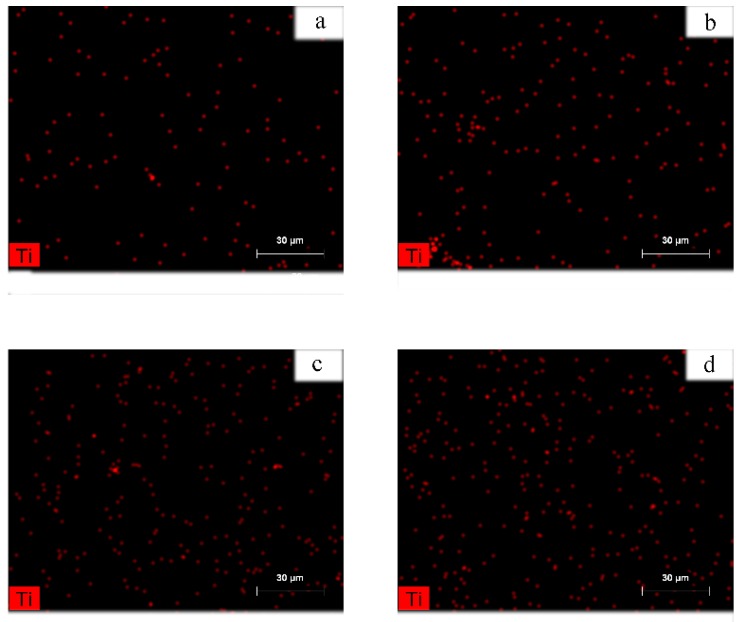
Energy-dispersive X-ray spectroscopy (EDS) mapping of the coPA films containing (**a**) 0.5, (**b**) 1.0, (**c**) 2.5 and (**d**) 5.0 wt. % of MXene.

Figure 5 shows the EDS spectra of the coPA/MXene composites. The detection of Ti (red) clearly demonstrates that even at high concentrations, the MXene particles have good dispersion within the coPA matrix, which is a requirement to utilize the MXene to modified polymeric matrix. The necklace like structure of the dispersed MXene particles within coPA matrix is observed, particularly at the lowest concentrations. This phenomenon might have considerable influence on the properties of composites in general.

#### 3.2.2. XRD Analysis of Composite Films

**Figure 6:**
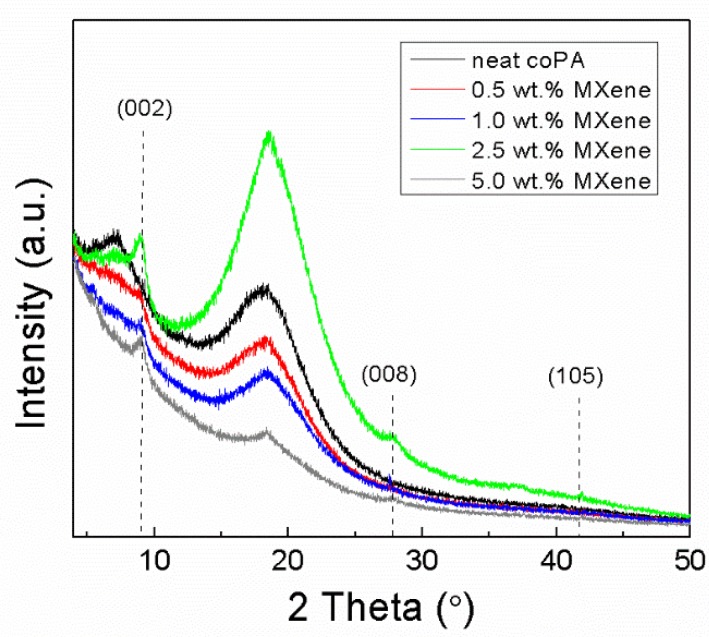
XRD spectra of the coPA/MXene films.

The XRD patterns of the neat coPA films and composites of coPA and MXene (up to 5 wt. % MXene filler) are shown in Figure 6. Noticeably, the XRD pattern of the neat coPA film confirms mostly the amorphous character of material, with low amounts of the crystalline phase. This study observed two main broad peaks at 2θ values of 7.1° and 18.2°. The main peak at 18.2° corresponds to the triclinic α-crystalline phase [51].

The composites containing MXene fillers show typical MXene peaks at 8.8°, 29° and 41.5° corresponding to (002), (008) and (105) planes, respectively. The typical MAX-phase peak for the (002) plane is at 9.5°. The shifting of the (002) peak to 8.8° is caused by the etching of Al from Ti_3_AlC_2_ and the subsequent replacement of Al by F, O and OH terminated groups from the Ti_3_C_2_T_x_ MXene structure, resulting in the expansion of *d*-spacing, as mentioned earlier. 

#### 3.2.3. Electrical Conductivity Measurements

First, the electrical conductivities of the as-prepared MXene and its MAX-phase precursor were determined and were found to be 9.1 S·cm^−1^ for MXene and 172 S·cm^−1^ for the MAX-phase. It is important to note that these values were obtained from the measurements on pellets, and therefore, they are far from the values reported for individual layers [52].

The investigation of pressed MXene flakes under various pressures and temperatures using fractions with various aspect ratios is currently in progress.

The electrical conductivity of the composites and the related percolation threshold were determined and investigated because these have potential applications as printed electronics or other conducting layers. As such, the electrical conductivity and the amount of filler are crucial parameters for further use of these materials. The percolation theory is commonly applied [52] to describe systems consisting of randomly dispersed conducting particles within an insulating matrix. The dependence of electrical conductivity on the volume filler content above the percolation concentration can be described by Equation (2) [52]:

(2) $$\sigma_{DC}\left(\phi \right)\propto {\left(\phi -\phi_{c}\right)}^{t}$$

where *ϕ* is the volume filler concentration, *ϕ_c_* is the percolation concentration, *σ_DC_* is DC conductivity estimated from low frequencies of AC conductivity, and *t* is the scaling exponent that characterizes the dimensionality of the investigated conductive system.

**Figure 7:**
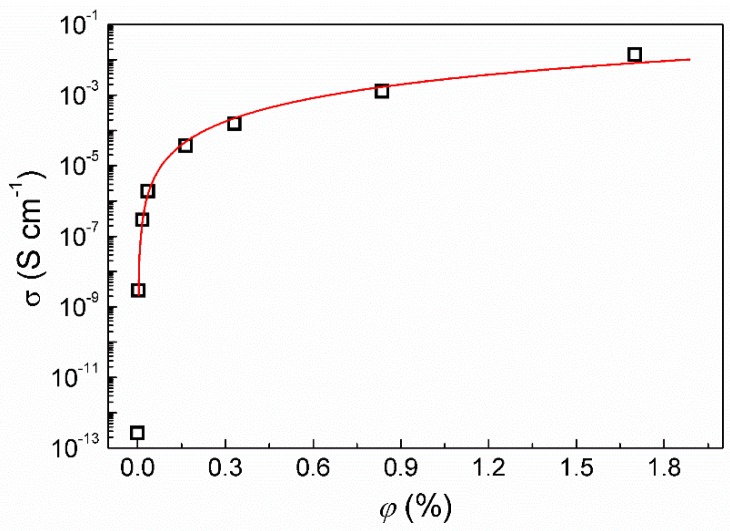
The dependence of the DC conductivity on the volume fraction for the various coPA/MXene composite thin films.

The dependence of the electrical conductivity on the filler content is shown in Figure 7. The conductivity of the neat coPA matrix is 3.5 × 10^−13^ S·cm^−1^, which agrees with other polyamide reports [53,54]. To determine the percolation concentration (*ϕ_c_*), the experimental data were fitted to Equation (1), which gave resultant values of *ϕ_c_* = 0.005 vol. % and *t* = 2.19, indicating a three-dimensional composite system.

For comparison, Kirkpatrick [55] calculated the following values for the critical exponent (*t*), *t* = 1.6 ± 0.1 (for the bond percolation model) and *t* = 1.5 ± 0.1 (for the point percolation model).

However, other critical exponents can also be found in the literature. According to Tchmutin et al. [56], the critical exponent for a three-dimensional system is *t* = 1.6–1.9, while the percolation concentration is 0.17 vol. %.

With respect to potential applications, because the low MXene content leads to a reasonable enhancement of the electrical conductivity and these suspensions can be easily applied by spraying, coating or spin-coating, these materials can be effectively used for low-cost antistatic or conductive coatings.

**Figure 8:**
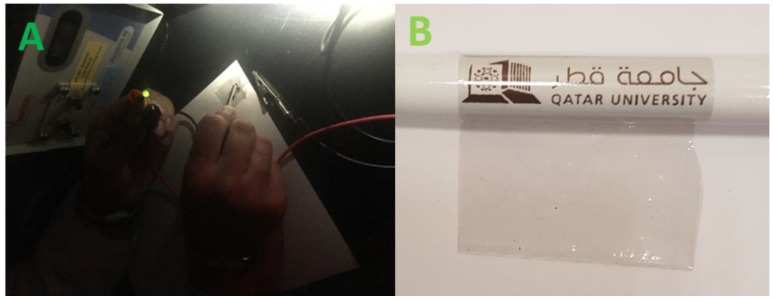
(**A**) The connection of coPA film containing 5 wt. % into electrical circuit. (**B**) coPA film containing 5 wt. % of MXene wrapped around a pencil.

The conductivity of the cast film is also illustrated in Figure 8A, showing that the applied voltage (2 V) successfully lights an LED connected into the composite film/wire/LED electrical circuit. Figure 8B demonstrates the high flexibility and transparency of the coPA film containing 5 wt. % of MXene wrapped around a pencil. 

#### 3.2.4. Dielectric Properties

**Figure 9:**
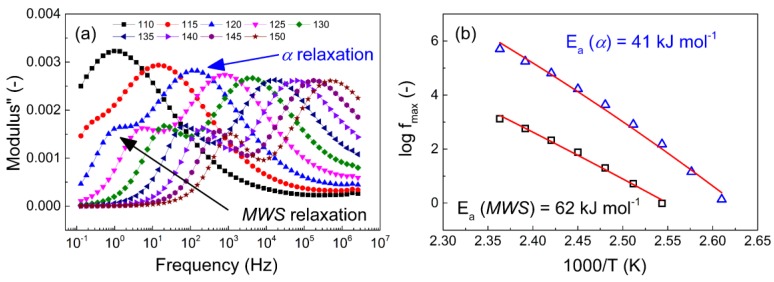
(**a**) The dielectric spectra of the neat coPA composite thin film at various temperatures and (**b**) the Vogel–Fulcher–Tammann plot of the two types of relaxations.

Figure 9 shows that the neat coPA exhibits two relaxations. The *α*-relaxation belongs to the main coPA polymer chain, while the secondary *β*-relaxation corresponds to the side groups of the polyamide. 

These relaxations are clearly visible only after recalculation of the dielectric loss factor to the loss modulus. This recalculation was based on Equation (3):

(3) $$\begin{array}{l} M^{*}=\frac{1}{\varepsilon^{*}}\\ M^{\prime }=\frac{\varepsilon^{\prime }}{{\varepsilon^{\prime }}^{2}+{\varepsilon^{\prime \prime }}^{2}}\\ M^{\prime \prime }=\frac{\varepsilon^{\prime \prime }}{{\varepsilon^{\prime }}^{2}+{\varepsilon^{\prime \prime }}^{2}} \end{array}$$

where ε* is the complex permittivity; *ε*′ and *ε*″ are the relative permittivity and dielectric loss factor, respectively; M* is the complex electric modulus; and *M*′ and *M*″ are the storage and electrical loss moduli, respectively.

The recalculation of the permittivities is necessary for conducting samples [57] or samples where the electrode polarization is very strong, similar to what was observed in other polyamide samples [58]. To quantify the activation energies of the main relaxations (*α* and Maxwell-Wagner-Sillars (MWS)), the Vogel-Fulcher-Tamman equation (Equation (4)) was effectively applied in a similar manner to various polymeric [57,59] or composite systems [60].

(4) $$f=f_{0}\exp\left(\frac{E_{a}}{k\left(T-T_{0}\right)}\right)$$

In Equation (3), *f* is the relaxation frequency, *f*_0_ is the pre-exponential factor, *E_a_* is the activation energy, *T* is the thermodynamic temperature, *T*_0_ is Vogel temperature, and *k* is the Boltzmann’s constant.

**Figure 10:**
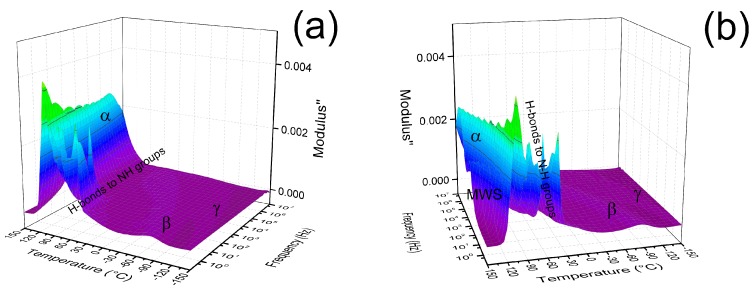
The dielectric maps for neat coPA matrix showing various types of relaxations, where (**a**) is neat coPA and (**b**) is neat coPa turned by 45° to right in order to highlight the MWS type of relaxation.

In this case, the semi-logarithmic plot shows linear behavior at various temperatures for the Arrhenius-like relaxation processes. Using this information, activation energies of 41 kJ·mol^−1^ and 62 kJ·mol^−1^ were found for the *α*-relaxation and *MWS*-relaxation, respectively. Moreover, from the dielectric map (Figure 10), it can be clearly seen that *β*-relaxation described as relaxations of the amide groups is visible at −60 °C. The further relaxation connected to the hydrogen bonding between the -OH and amide groups can be seen from 75 °C, while *γ*-relaxation, which is connected to the CH_2_ groups in case polyamides are present and in case of more than (CH_2_)_4_ in the polymer backbone which was observed at −120 °C, similarly as was observed in several studies [54,61].

To investigate the effect of the presence of MXenes on the polyamide chain dynamics, similar data processing was used. However, since nearly all of the investigated samples were above the percolation threshold, there were no relaxation peaks in the broad temperature range from −150 to 150 °C. Therefore, further investigation was not possible.

#### 3.2.5. Charge Transport Mechanism

**Figure 11:**
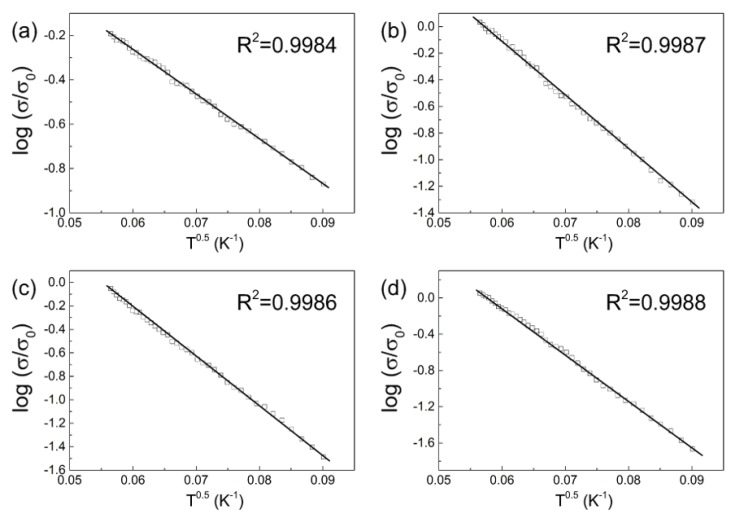
The fitting of experimental data by the Mott equation for neat coPA and coPA/MXene composite films: (**a**) neat coPA, (**b**) 1.0, (**c**) 2.5 and (**d**) 5 wt. % of MXene.

Since the percolation threshold was found to be very low, mainly due to the presence of the hydrophilic MXene and its conductivity and filler dispersion, the charge transport of the composites was investigated. Similarly to reports in the literature [57,58,62,63], the conductivity mechanism can be described by the Mott Equation (5) as shown in Figure 11:

(5) $$\sigma \left(T\right)=\sigma_{0}\exp\left(-{\left(\frac{T_{0}}{T}\right)}^{n}\right)$$

where *n* is the dimensionality of charge transfer, *T*_0_ is the Mott characteristic temperature of the insulator-to-metal transition, and *σ*_0_ is the conductivity at ambient temperature. 

**Figure A1:**
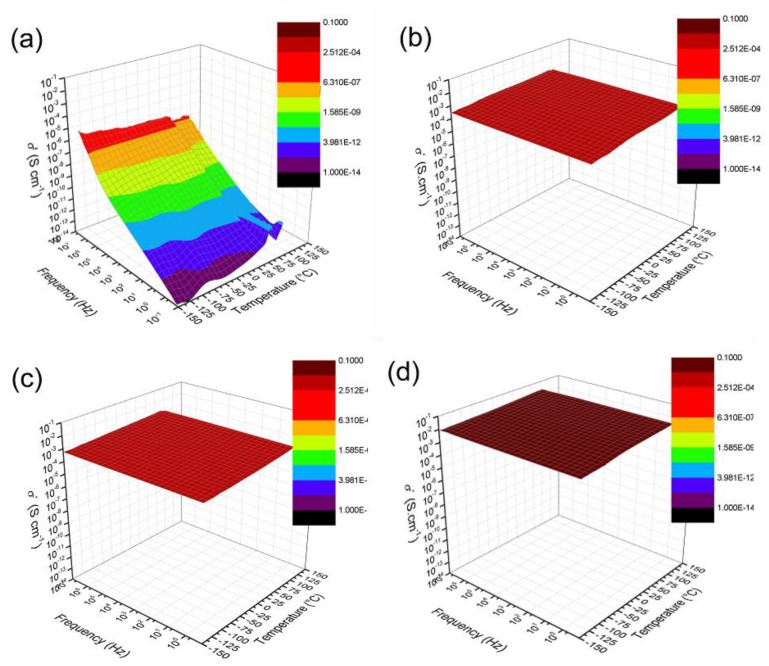
Dielectric maps of coPA/MXene composite films: (**a**) neat coPA, (**b**) 1.0, (**c**) 2.5 and (**d**) 5 wt % of MXene.

The neat coPA matrix exhibited typical insulator behavior of considerably increased AC conductivity with increasing frequency and temperature (Figure A1). On the other hand, the rest of the investigated composites have conductivities above the percolation threshold and do not exhibit a typical metal-to insulator transition (MIT). Thus, fitting with Equation (4) should shed light on the mechanism of charge transport in the coPA/MXene composites. For all composite samples, the *n* parameter reaches 0.5, and thus, a tunneling mechanism between the thin insulating barriers was observed, similar to that found in various polypyrrole-based nanostructures with similar DC conductivities [64].

#### 3.2.6. UV/Vis Characterization

**Figure 12:**
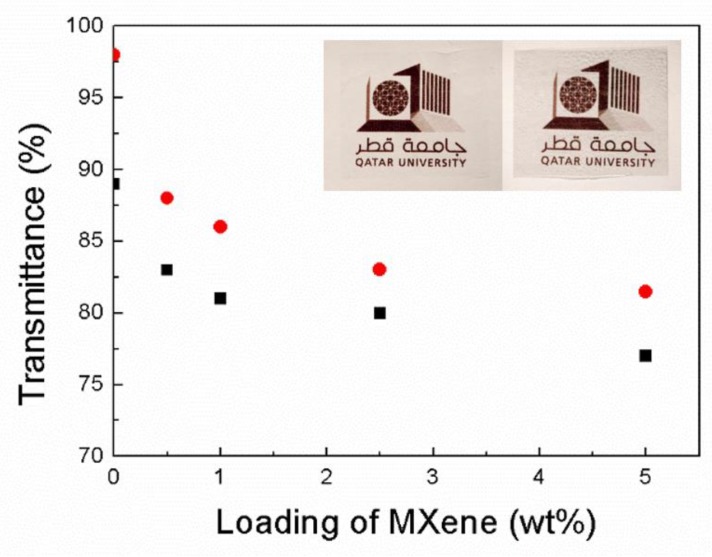
Transmittance of the coPA/MXene films at 550 nm (black square) and 650 nm (red circles) with an inserted picture of the QU logo covered by neat coPA (**left**) and coPA filled with 5 wt. % of MXene (**right**). The thickness of both films is approximately 150 µm.

The transparency of the coPA/MXene films at 550 and 650 nm is shown in Figure 12. The transparency of the film decreased with the increasing loading of MXene. due to the chemical nature of the MXene filler. However, even at an MXene filler loading of 5 wt. %, a transparency over 75% was observed at both wavelengths.

The good transparency of the neat coPA and the coPA/MXene composites is also shown in the inserted picture of the QU logo covered with neat coPA and coPA film containing 5 wt. % of MXene in Figure 12.

#### 3.2.7. Dynamic Mechanical Analysis

**Figure 13:**
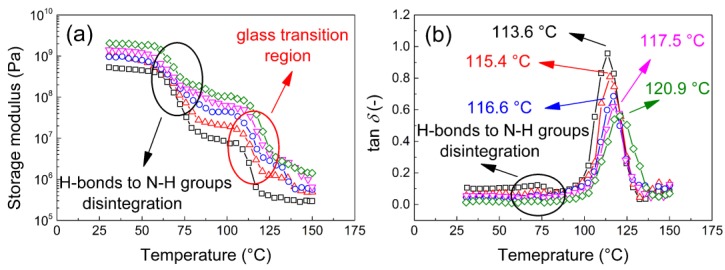
Temperature dependence of the (**a**) storage modulus and (**b**) tan δ of various composite thin films, including neat coPA (black squares), 0.5 wt. % MXene composite (red triangles), 1 wt. % MXene composite (blue circles), 2.5 wt. % MXene composite (magenta downward triangles) and 5 wt. % MXene composite (green diamonds).

The suitable mechanical properties are very important for the application of these materials. The polyamide used in this work is a hot-melt type and thus can be easily applied at moderate temperatures or sprayed from solution, as it has very good solubility in the common, low-cost and environmentally friendly solvent n-propanol. Therefore, after the deposition of a compact layer or coating on a solid substrate, measuring the mechanical and/or dynamic mechanical properties of these materials is important for their final application. Figure 13 clearly shows that the storage moduli increase with increasing MXene content, indicating excellent dispersion of the filler within the polymer matrix. 

**Figure 14:**
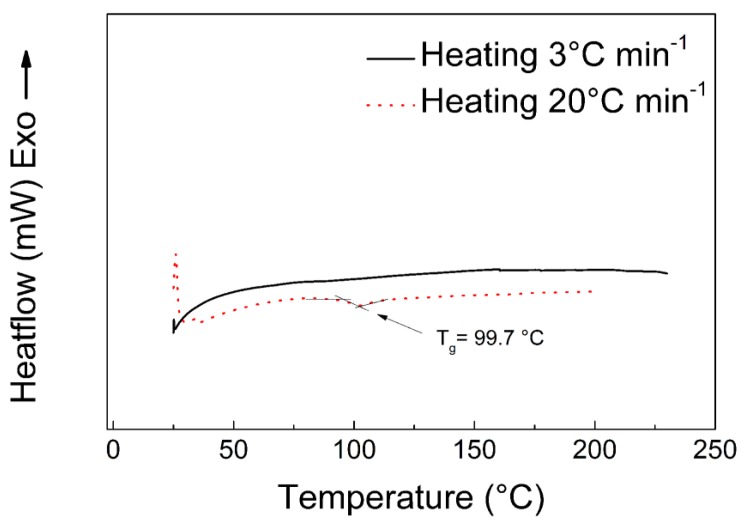
DSC spectra for neat coPA.

All samples exhibit two transitions, and the first one is a visible decrease in the storage modulus from 10^9^ Pa to 10^7^ Pa from 60 to 80 °C. This drop is the greatest in the neat coPA and it is associated with the disintegration of the H-bonds between the OH groups and amide groups of copolyamide, while the composites show a suppressed transition (peak of the tan*δ* at 75 °C nearly disappeared in Figure 13b) but visible in Figure 13a (the drop in the storage modulus). The second transition from 110 to 130 °C corresponds to the glass transition, and the final values of the storage modulus reach 10^5^ Pa. The presence of *T_g_* in this region was confirmed also by DSC measurements (Figure 14), when the *T_g_* is clearly visible for the rapid heating (20 °C/min). In order to be sure that this region is not connected to the melting of the developed crystallites in the sample, in DMA the melting can be also showed as the tan*δ* peak [65]. The sample was investigated in a whole temperature range at a slow heating rate (3 °C/min), providing the suitable time for developing of the crystalline phase. It is clear from Figure 14, that the presented sample is amorphous. The assigned *T_g_* is also clearly visible in dielectric maps (Figure 10) in the region from 90 to 120 °C depending on the applied frequency of the electric field. As the amount of MXene in the composites increased, the mechanical properties above the *T_g_* increase, and with 5 wt. % of MXene, the storage modulus reaches 10^6^ Pa. This finding confirms that effective dispersion of the filler improves the mechanical properties of these materials in comparison to other investigated systems [66,67]. This reinforcing effect can also be observed in Figure 13b, where the tan*δ* peak increases from 113.6 to 120.9 °C for neat coPA and the 5 wt. % MXene composite, respectively.

To quantify the performance of these materials, the pseudo-activation energies were calculated according to the Arrhenius theory and a modified equation, which was used as in other reports [68,69]:

(6) $$\ln f=\ln f_{0}-\frac{E_{a}}{RT_{g}}$$

where *f* is the tested frequency, *f*_0_ represents the characteristic constant of the material, *T_g_* is the glass transition temperature, and *R* is the universal gas constant.

The filler-matrix interaction can be quantified as an energetically activated barrier, which must be overcome during mechanical stimulation. Therefore, the frequency dependence of the *T_g_* was plotted, and the calculated activation energies are summarized in Table 2. With an increasing amount of filler, the composites show higher activation energies and thus excellent filler-matrix interactions. There is a significant enhancement of the activation energy from 373 to 505 kJ·mol^−1^ for neat coPA and the 5 wt. % MXene composite, respectively. This result is caused by a combination of two factors: The good mechanical properties of the neat filler; the good compatibility between the hydrophilic filler and the hydrophilic matrix. This provides excellent properties in the final composite thin films.

## 4. Conclusions

The successful preparation and characterization of electrically conductive, highly transparent and flexible self-standing thin films based on coPA-6,10 filled with up to 5 wt. % of MXene nanosheets prepared by casting is reported.

The MXene nanosheets were isolated from a MAX phase (Ti_3_AlC_2_) using an HF solution and their nanoscale dimensions were confirmed by TEM and AFM measurements.

The percolation threshold of the MXene filler within the coPA matrix was found to be 0.05 vol. %, and the highest determined electrical conductivity was 1.4 × 10^−2^ S·cm^−1^ for composites filled with 5 wt. % (1.8 vol. %) of MXene.

The transparency of the prepared films exceeded 75%, even for samples containing 5 wt. % of MXene, as confirmed by UV spectroscopy.

An investigation of the dynamic mechanical properties indicated enhanced filler-matrix interactions, supported by the calculated activation energy reaching 500 kJ·mol^−1^ for coPA modified with 5 wt. % of MXene. The mechanical properties, such as the storage modulus, were improved by increasing the amount of MXene.

The combination of the relatively high electrical conductivity of the composites with low filler content, appropriate transparency, and good mechanical properties make these materials promising for applications in flexible electronics.

## Figures and Tables

**Table 1:** The apparent surface chemical composition as determined by X-ray photoelectron spectroscopy (XPS).

Sample	Surface Chemical Composition [at. %]					
	C1s carbide/sp^2^/sp^3^/CO/OC=O	O1s oxide/C=O/CO	Ti2p TiC/Ti^2+^/Ti^3+^/TiO_2_	Al2p Al/AlO_x_	F1s F^−^/X-F/X-F-X	N1s
MAX phase	33.5 5.2/3.3/19.8/2.8/2.4	38.2 16.1/14.5/7.5	11.6 4.4/1.5/1.2/4.6	12.0 2.1/9.9	3.0 0.8/1.6/0.6	1.8
MXene	42.9 9.6/9.4/14.9/6.1/2.9	19.7 8.1/6.9/4.7	18.3 6.8/5.3/2.6/3.6	3.8 0.8/3.0	13.5 2.5/7.6/3.4	2.0

**Table 2:** The calculated activation energies for various composite thin films.

Sample Composition	*T_g_* Values of coPA/MXene Films (°C)				*E_a_* (kJ/mol)
	0.5 Hz	1 Hz	2.5 Hz	5 Hz	
neat coPA	112.0	113.6	114.4	115.4	373
0.5 wt. % MXene	114.3	115.4	116.3	117.5	405
1 wt. % MXene	115.5	116.6	117.3	118.6	423
2.5 wt. % MXene	116.5	117.5	118.4	119.3	464
5 wt. % MXene	120.1	120.9	121.8	122.7	505
